# Case Report: First Confirmed Case of Coinfection of SARS-CoV-2 With *Choclo orthohantavirus*

**DOI:** 10.3389/fitd.2021.769330

**Published:** 2021-11-10

**Authors:** Susana Hesse, Heydy Nuñez, Jacqueline R. Salazar, Tybbysay P. Salinas, Erika Barrera, Ricardo Chong, Saúl Torres, Amarellys Cumbrera, Idiamín Olivares, Aimee Junco, Christian Matteo, Claudia González, Oris Chavarría, Ambar Moreno, Jessica Góndola, Leyda Ábrego, Yamilka Díaz, Yaneth Pitti, Danilo Franco, Mabel Martínez-Montero, Juan Miguel Pascale, Sandra López-Vergès, Alexander A. Martínez, Blas Armién

**Affiliations:** ^1^ Intensive Care Unit, Luis “Chicho” Fabrega Hospital, Ministry of Health, Santiago, Panama; ^2^ Department of Epidemiology, National Social Security Fund, Santiago, Panama; ^3^ Department of Epidemiology, Ezequiel Abadia Hospital, Soná, Panama; ^4^ Department of Research in Emerging and Zoonotic Infectious Diseases, Gorgas Memorial Institute for Health Studies, Panama City, Panama; ^5^ Regional Department of Epidemiology, Ministry of Health, Santiago, Panama; ^6^ Department of Genomics and Proteomics, Gorgas Memorial Institute for Health Studies, Panama City, Panama; ^7^ Department of Research in Virology and Biotechnology, Gorgas Memorial Institute for Health Studies, Panama City, Panama; ^8^ National Research System (SNI), National Secretary of Research, Technology and Innovation (SENACYT), Panama City, Panama; ^9^ Inmunovirology Section, Central Reference Laboratory in Public Health, Gorgas Memorial Institute for Health Studies, Panama City, Panama

**Keywords:** *SARS-CoV-2*, *Choclo orthohantavirus*, COVID-19, hantavirus pulmonary syndrome, coinfection, case report

## Abstract

The emergence of severe acute respiratory syndrome coronavirus 2 (SARS-CoV-2) has caused a major international public health concern. The World Health Organization (WHO) declared the pandemic of coronavirus disease 2019 (COVID-19) on March 11, 2020. In Panama, the first SARS-CoV-2 infection was confirmed on March 9, 2020, and the first fatal case associated to COVID-19 was reported on March 10. This report presents the case of a 44-year-old female who arrived at the hospital with a respiratory failure, five days after the first fatal COVID-19 case, and who was living in a region where hantavirus pulmonary syndrome cases caused by *Choclo orthohantavirus* (CHOV), are prevalent. Thus, the clinical personnel set a differential diagnosis to determine a respiratory disease caused by the endemic CHOV or the new pandemic SARS-CoV-2. This case investigation describes the first coinfection by SARS-CoV-2 and CHOV worldwide. PCR detected both viruses during early stages of the disease and the genomic sequences were obtained. The presence of antibodies was determined during the patient’s hospitalization. After 23 days at the intensive care unit, the patient survived with no sequelae, and antibodies against CHOV and SARS-CoV-2 were still detectable 12 months after the disease. The detection of the coinfection in this patient highlights the importance, during a pandemic, of complementing the testing and diagnosis of the emergent agent, SARS-CoV-2, with other common endemic respiratory pathogens and other zoonotic pathogens, like CHOV, in regions where they are of public health concern.

## Introduction

Coronavirus disease 2019 (COVID-19), caused by the severe acute respiratory syndrome coronavirus 2 (SARS-CoV-2), emerged in Wuhan, China, at the end of 2019. The clinical profile is characterized by a short incubation period and prodromal symptoms as fever and cough. After 3 to 6 days of symptoms, severe cases may evolve to dyspnea, pneumonia, and respiratory failure (1). SARS-CoV-2, transmitted from person to person through aerosols (2), spread around the world and was declared a pandemic by World Health Organization (WHO) on March 11, 2020.

In Panama, the first confirmed case of SARS-CoV-2 was reported on March 9, 2020, with the first fatal case reported one day after, on March 10. The epidemiological and genomic analysis of SARS-CoV-2 at the beginning of the epidemic in Panama, suggested that many lineages were introduced during February 2020. These variants circulated undetected and included the emergence of an endemic variant (3). The rapid increase of positive SARS-CoV-2 cases made the clinical and laboratory response focus on the COVID-19 pandemic. However, other pathogens continued causing disease in Panama. Pathogens that cause symptoms similar to SARS-CoV-2, if differential diagnoses are not taken into account, could add to misdiagnosis and un-detection of possible coinfections. Moreover, coinfections could be of clinical concern due to the possible increase of morbidity and mortality, especially in low- and middle-income countries. For example, cases of coinfection of SARS-CoV-2 and dengue in Asia (4) and in the Americas (5) have been reported, as well as coinfection of SARS-CoV-2 with other tropical pathogens like *Orientia tsutsugamushi*, causing scrub typhus, a public health concern in Asian countries. In Panama, dengue (6) as well as other arboviral and zoonotic diseases, are endemic.

In central Panama, hantavirus pulmonary syndrome (HPS) is prevalent. HPS is caused by *Choclo orthohantavirus* (CHOV), which emerged in 2000 and now is endemic (seroprevalence of 26%) in the central region (**Supplementary Figure 1**) (7–10). HPS is mainly transmitted by inhalation of aerosols contaminated with rodent excreta, as the pigmy rice rat *Oligoryzomys fulvescens* (=*costaricensis*) is the rodent species reservoir of CHOV (11, 12). After infection, CHOV has an incubation period between 2 to 6 weeks, with a prodromic phase that usually lasts no more than five days. The appearance of cough and tachypnea can develop into respiratory failure (13). The prodromal phase and respiratory failure caused by SARS-CoV-2 and CHOV are indistinguishable through the clinical eye, translating into a challenging diagnosis (14).

Here, we report the epidemiological, clinical, and laboratory characteristics of a case with coinfection of SARS-CoV-2 and CHOV at the beginning of the COVID-19 pandemic in Panama, as the first reported coinfection between coronavirus and orthohantavirus worldwide. This case emphasizes, that the timely laboratory diagnosis of SARS-CoV-2 infection should complement the analysis for endemic or co-circulating pathogens that cause similar symptoms. Furthermore, this simultaneous laboratory analysis could detect possible coinfections for an accurate diagnosis and a better management of patients.

## Case Presentation

**Figure 1:**
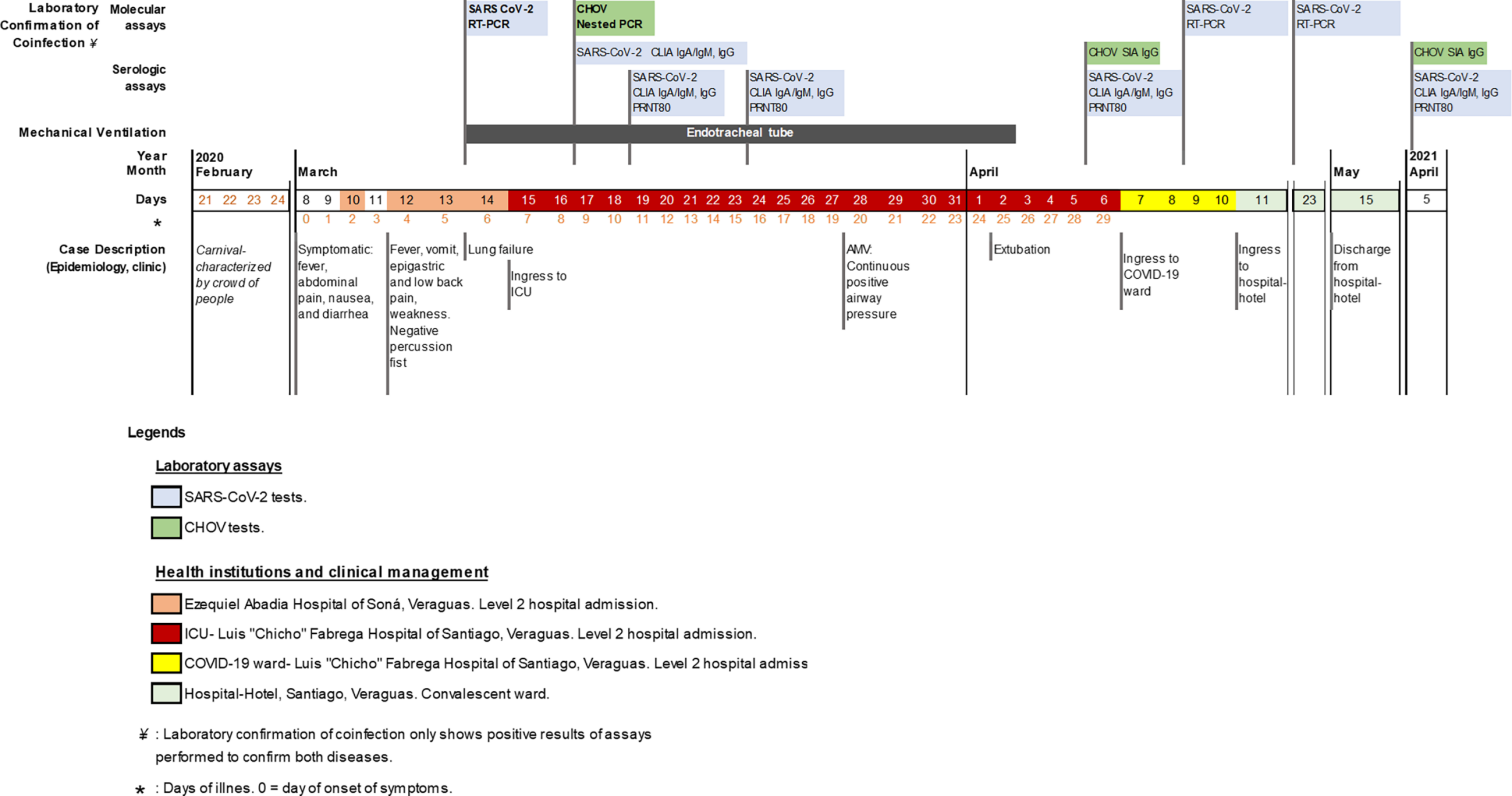
Timeline: Clinical, epidemiological and laboratory assays of SARS-CoV-2 and CHOV coinfection case. Schematic timeline with events described from top to bottom: molecular and serological laboratory assays for SARS-CoV-2 (light blue) and CHOV (green), type of mechanical ventilation applied to the patient, timeline with the date (year, month, day) and the days of symptoms onset (*) with color coding representing the health institution and clinical management, epidemiological and clinical case description. Abbreviations used in the figure: SARS-CoV-2, severe acute respiratory syndrome coronavirus 2; CHOV, *Choclo orthohantavirus*; RT-PCR, real time reverse transcription polymerase chain reaction; PCR, polymerase chain reaction; CLIA, chemiluminescent immunoassay; SIA, strip immunoblot assay; PRNT80, 80% plaque reduction neutralization test; Ig, immunoglobulin (A, M and G for this case); ICU, intensive care unit; AMV, advance in mechanical ventilation; COVID-19, coronavirus disease 2019.

**Figure 2:**
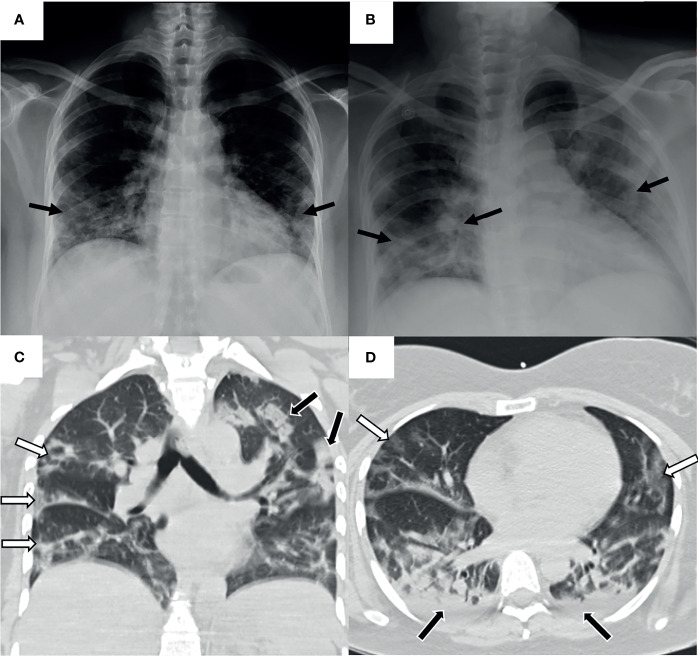
Pulmonary images of the patient with HPS and COVID-19. Taken in the intern medicine ward. **(A)** Posteroanterior chest radiography. Mild peripheral diffuse infiltrate of predominance in both pulmonary bases (black arrows). No cardiomegaly, no pleural effusion. **(B)** Anteroposterior chest radiography. Increased extent of the radiographic infiltrate in both pulmonary fields (black arrows). No pleural effusion. A rapid progression of the infiltrate is observed in 10 hours of evolution compared to previous radiography. **(C)** Coronal chest high-resolution simple computed tomography. Scattered multifocal opacities of peripheral predominance in both lungs (white arrows). Isolated consolidated areas (black arrows). **(D)** Axial chest high-resolution simple computed tomography. Tarnished glass-like opacities in anterior pulmonary location (white arrows) and consolidated areas in posterior pulmonary location (black arrows). Mild bilateral basal posterior pleural effusion (black arrows).

A 44-year-old female patient from, province of Veraguas, in central Panama, presenting with obesity, yet without other pathological antecedents. On March 8, 2020, the patient reported gastrointestinal symptoms (vomit and diarrhea) accompanied by unquantified fever of 9 days of evolution (**Figure 1**). After visiting the emergency room of the secondary care hospital (Ezequiel Abadia Hospital), the patient received symptomatic outpatient treatment. On March 10, 2020, urinalysis presented with leukocyturia and bacteriuria; she was diagnosed with a urinary tract infection, managed with ciprofloxacin, acetaminophen, and recommendations for outpatient care. Two days later, on March 12, due to the persistence of the symptoms, the patient was admitted to the same hospital for observation. Chest radiography, hematology test, and Dengue virus serology test were performed with no relevant findings. On March 13, the patient presented stable vital signs, temperature (T)=36.8°C, blood pressure (BP)=121/81 mmHg, heart rate (HR)=96 bpm and respiration rate (RR)=18 rpm. Eight hours later, the patient started a persistent cough, tachypnea (RR=36 rpm), and arterial oxygen saturation of SaO_2_=93% (**Table 1**). The posteroanterior chest radiography showed a mild peripheral diffuse infiltrate with predominance in both pulmonary bases (**Figure 2A**). Wherefore, oxygen therapy with nasal cannula was started early in the morning on March 14. The same day in the evening, the anteroposterior chest radiography showed an increased extent of the infiltrate in both pulmonary fields (**Figure 2B**), with worsening clinical conditions. The arterial gasometry showed SaO_2_=96%, arterial partial pressure of oxygen (pO_2_)=72 mmHg and standard bicarbonate (sHCO_3_)=23.9 mmol/L, arterial partial pressure/fraction of inspired oxygen (PaO_2_/FiO_2_)=180. The patient required intubation and mechanical ventilation and was transferred, to the secondary care hospital, Luis “Chicho” Fabrega Hospital. After evaluation by the Pneumology Service in the emergency room, the patient was admitted directly to the intensive care unit (ICU). A high-resolution chest computed tomography reported scattered multifocal opacities of peripheral bilateral predominance with ground glass images, consolidative foci, and scarce bilateral pleural effusion (**Figures 2C, D**). The patient was hemodynamically compensated with vasopressor support and maintained under sedoanalgesia coupled to mechanical ventilation in assisted-control pressure mode. The patient had feverish peaks that persisted during the following days. The patient presented transaminases slightly increased (AST range: 34-74U/L; ALT range: 30-74U/L). However, she did not present thrombocytopenia (Platelets range: 154-740x10^3^) nor hemoconcentration (Hematocrit range: 24.9-37.2%). Other clinical laboratory analyses showed no relevant findings (**Table 1**).

**Table 1:** Vital signs and clinical laboratory analysis during emergency and hospitalization.

Clinical laboratory	Ezequiel Abadia Hospital			Luis “Chicho” Fabrega Hospital						Reference values
	Emergency	Observation	Intern Medicine Ward	ICU Ingress	Diminution of PEEP	Failed CPAP	Advance CPAP	ICU Egress	Egress to ward	
** **	**3/10/2020**	**3/12/2020**	**3/14/2020**	**3/15/2020**	**3/25/2020**	**3/26/2020**	**3/28/2020**	**4/6/2020**	**4/10/2020**	** **
**Vital signs**	** **	** **	** **	** **	** **	** **	** **	** **	** **	** **
BP (mm Hg)	120/80	110/80	129/83	142/88	110/63	118/70	107/63	125/92		
HR (bpm)	78	80	105	87	101	73	110	82		
RR (rpm)	20	18	36	24	14	23	20	18		
Pulse Oximetry (%)			96	100	97	97	97	100		
Temperature (Celsius)	37°	>38°	38°	37°	37°	37.5°	36.9°	37.6°		
Weight (kg)		82		81.5	83	83	85.5	78		
				** **						
**Hemogram**	** **	** **	** **	** **	** **	** **	** **	** **	** **	** **
Hemoglobin (g/dL)		12.7	11.9	11.7	9	8.1	8.1	9.1	9.7	12.0-16.0
Hematocrit (%)		37.2	34.3	35.2	27.6	24.9	25.7	28.2	29	36-48
WBCs (x10^3^)		4.07	6.84	14	13	14.3	7.6	10	7.5	4.0-10.0
Neutrophils (%)		79.1	86.3	79.4	77.9	84.2	68.7	66.3	59.3	50-80
Lymphocytes (%)		0.7	0.7	18.6	11.9	7.9	18.8	23.5	29.2	17-40
Eosinophils (%)		0	0	0	1.3	1.5	3.1	3.3	4.1	0.9-6.0
Platelets (x10^3^)		158	154	216	676	667	740	602	364	140-400
										
**Chemistry**	** **	** **	** **	** **	** **	** **	** **	** **	** **	** **
Glucose (mg/dL)				127	148	172	118	105	126	74-106
Creatinine (mg/dL)			0.84	0.6	0.5	0.5	0.5	0.4	0.4	0.7-1.2
BUN (mg/dL)			0.71	5	13	18	13	7	6	7.0-17.0
Na (mmol/L)			142	144	142	144	144	142	144	137-145
K^+^ (mmol/L)			3.3	3.4	4.1	4.5	5	4.6	3.8	3.5-5.1
Cl^-^ (mmol/L)				112	102	107	109	106	108	98-107
Ca^2+^ (mg/dL)				8	9	8.6	9	10.1		8.4-10.2
P^3+^ (mg/dL)				2.7	4.7	3.2	3.7	3.3		2.5-4.5
Mg^2+ ^ (mg/dL)				2	2.1	2.4	2.2	1.9		1.6-2.3
GGT (U/L)				193				65		12.0-43.0
AST (U/L)		50		74		49		35	34	14.0-36.0
ALT (U/L)		30		40		74		53	55	9.0-52.0
LDH (U/L)		380	440	594						313-618
Albumin (g/dL)			3.7	3.5	4 (03/24/20)			4.4		3.5-5.0
Total bilirubin (mg/dL)				0.8						0.2-1.3
DBIL/IBIL (mg/dL)				0.30/0.50						0.0-0.3/0.0-1.1
ALP (U/L)		Normal		94						38.0-126.0
Amilase (U/L)		105								28.0-100.0
PCR (mg/dL)			13.7							0.0-10.0
Procalcitonin (ng/mL)			<0.05							0.0-0.05
										
**Urinalysis**										
Bacteriuria	+	–								Negative
Urine leukocyte count (/hpf)	+	36								0-5
Urine erythrocyte count (/hpf)		19								0-5
										
**Arterial blood gas**	** **	** **	** **	** **	** **	** **	** **	** **	** **	** **
pH			7.54	7.48	7.39	7.43	7.43	7.56		7.35-7.45
PaCO_2_ (mmHg)			28	27	43	43	43	28		35-45
PaO_2_ (mmHg)			72	203	124	118	103	202		60-100
sHCO_3_ (mmol/L)			23.9	20.1	27.1	27.9	29.2	25.1		22-26
EB (mmol/L)			1.4	-3.4	2.1	4.2	4.9	2.9		±2.0
SaO_2 _ (%)			96	100	98	99	98	100		94-99
Lactate (mmol/L)			0.8	0.9	1.8	0.9	0.7	0.9		0.5-1.0
Oxygen therapy			NC	MV	MV	MV	MV	NC		
FiO_2_ %			40	50	35	30	30	28		
										
**Coagulation test**										
PT (sec)			13.9	14.1						11.3-14.5
PTT (sec)			29.2	27.2						24-34
INR			1.02	1.12						0.8-1.2
Fibrinogen (mg/dL)			401	559						200-400

ICU, Intensive care unit; PEEP, Positive End-Expiratory Pressure; CPAP/MV, Continuous positive airway pressure/mechanical ventilation; BP, Blood pressure; HR, Hearth rate; RR, Respiratory rate; WBCs, White blood cells; BUN, Blood urea nitrogen; GGT, Gamma-glutamyl transferase; AST, Aspartate aminotransferase; ALT, Alanine transaminase; LDH, Lactate Dehydrogenase; TBIL, total bilirubin; DBIL, Direct bilirubin; IBIL, Indirect bilirubin; ALP, Alkaline phosphatase; CRP, C-reactive protein; PaCO_2_, Arterial partial pressure of carbon dioxide; PaO_2_, Arterial partial pressure of oxygen; sHCO_3_, Standard bicarbonate; EB, Excess base; SaO_2_, Arterial oxygen saturation; Oxygen therapy= NC, Nasal cannula, MV, Mechanical ventilation; FiO_2_, Fraction of inspired oxygen; PT, Prothrombin time; PTT, Partial thromboplastin time; INR, International normalized ratio.

Once in ICU, the patient was managed as a presumptive case of HPS, considering the clinical symptoms and that the patient lived in an endemic region for CHOV (**Supplementary Figure 1**) and reported possible contact with the reservoir *Oligoryzomys costaricensis*. As Panama already had confirmed cases of COVID-19 since March 9, the clinical personnel took nasopharyngeal swab and sera samples for SARS-CoV-2 and CHOV laboratory diagnosis, respectively (**Supplementary Material and Methods**). The molecular tests confirmed the coinfection by SARS-CoV-2 6 days after symptom onset (first positive RT-PCR on March 14) and then by CHOV (positive Nested PCR on March 17) (**Figure 1**).

The patient developed specific antibodies against SARS-CoV-2, which were detected by chemiluminescent immunoassay (IgA/IgM >0.6 and IgG >1.6 cutoff) on March 17, with antibody increase during the next 20 days. The neutralization activity of these antibodies was analyzed by plaque reduction neutralization test (PRNT), and was detected on March 19, five days only after SARS-CoV-2 real time reverse transcription polymerase chain reaction (RT-PCR) detection. For antibodies detection against CHOV, enzyme immunoassay (EIA; Focus Diagnostics) and strip immunoblot assay (SIA) (15) were performed. IgM by EIA was not detectable (IgM >1.1 cutoff), neither by SIA. IgG by SIA was detected on April 6. The patient took longer to produce antibodies against CHOV; indeed, specific IgG were detected 20 days after its Nested PCR confirmation (**Figure 1** and **Supplementary Table 1**).

Since her admission, antibiotic therapy was given with levofloxacin (750 mg intravenous/day, for 10 days) and piperacillin/tazobactam (4.5 g/six hour, for 17 days), in addition, after SARS-CoV-2 laboratory confirmation, hydroxychloroquine (400mg/day, for 9 days) was started on March 16, according to national the treatment protocol used at that time for COVID-19 (**Figure 1**) (16). The patient did not develop liver, kidney, or cardiovascular failure. With favorable clinical, gasometrical, and radiological evolution, the ventilatory support was diminished to minimum requirements on the following days. On March 28, the weaning from sedation was started, and the advancement to spontaneous mode continuous positive airway pressure with pressure support (CPAP+PS) was made with good tolerance. After analyzing the clinical variables and respiratory maneuvers, we proceeded with the endotracheal extubating protocol on April 2. Oxygen therapy with a partial rebreathing reservoir mask FiO_2_ 60% was well tolerated. She continued hemodynamically stable, without increased respiratory effort and SaO_2_=98-99%, with adequate oxygenation and good gasometrical control (Kirby index or PaO_2_/FiO_2_=348). This evolution led to reduced oxygen supply, and it was decided to transfer the patient to the women’s COVID-19 ward on April 6 (**Figure 1** and **Table 1**).

During the patient´s hospitalization in the ward, the patient remained without changes in her condition, tolerating ambient air, wandering, with oral diet, afebrile, hemodynamically stable, and without respiratory distress. A control nasopharyngeal swab and PCR for SARS-CoV-2 was performed on April 9, before hospital discharge. However, the patient persisted positive by PCR. After completing antibiotic therapy, the patient was transferred to a nearby hospital-hotel in Santiago on April 10. The next PCR-control swab was performed on April 23, persisted positive; the patient persisted in the hospital-hotel isolation room. Other swabs were repeated on May 7 and 11, both resulted negative, therefore the patient was discharged from the hospital-hotel on May 15, after 62 intra-hospital days (**Figure 1**). During the one-year follow-up, the patient had no sequelae and still has detectable antibodies against SARS-CoV-2 and CHOV and antibody neutralization activity against SARS-CoV-2 (**Figure 1** and **Supplementary Table 1**).

**Figure 3:**
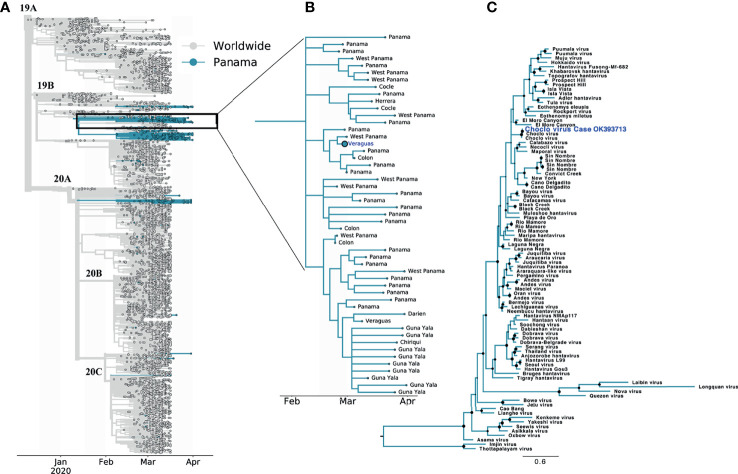
Phylogenetic trees of the SARS-CoV-2 and CHOV sequences from Panama and worldwide. **(A)** SARS-CoV-2 phylogenetic tree showing 6610 genomes sampled between December 2019 and April 2020, the analysis grouping the sequences in clades (name indicated at the base of each clade branch) was restricted to samples submitted to GISAID until October 2020 (grey: worldwide, blue: Panama). **(B)** SARS-CoV-2 subtree showing samples from the 19B clade that forms the cluster of transmission associated to the coinfection case (bigger circle with the sample location in blue). The sample locations at the level of health region within Panama are indicated. For acknowledgment table see **Supplementary Data**. **(C)** Phylogenetic tree of the orthohantavirus, including CHOV sequence with GenBank accession number OK393713 (blue) with. Maximum likelihood tree was drawn using worldwide reference sequences of orthohantavirus and rooted to Imji virus reference strain, black circles indicates nodes with ultrabootstrap values greater the 50%. All information about GISAID and Genbank accession numbers for the SARS-Cov-2 and orthohantavirus reference sequences, respectively, are listed in **Supplementary Data**.

Both viruses were sequenced to complete the molecular diagnosis and genetically characterize the viruses (**Supplementary Material and Methods**). The SARS-CoV-2 complete genome was sequenced to perform phylogenetic analyses (GISAID: EPI_ISL_1502818). The phylogenetic tree revealed that early in the pandemic sequences from Panama were grouped in different clades, suggesting several introductions of the virus during this period. Even if various clades were co- circulating, most of the initial cases were caused by the 19B clade-related sequences, in contrast with the rest of the world, where the dominant clades were 20A and 20B (**Figure 3A** and **Supplementary Table 2**) (3). Additionally, the genome obtained from this coinfection case grouped with SARS-CoV-2 samples collected from distant areas of the country, suggesting that the virus was already disseminated through Panama territory (**Figure 3B**). The sequencing of the small segment (S segment) of CHOV (GenBank: OK393713), which encodes the N protein, confirmed that the virus responsible for this case belongs to the same clade of the endemic virus previously reported in Panama (**Figure 3C** and **Supplementary Table 3**) (9, 17).

## Discussion

This clinical case documented in Panama is the first confirmed coinfection between a coronavirus, SARS-CoV-2, and an orthohantavirus, CHOV, worldwide, according to the definition of coinfection established by the CDC (18). The central-west region of the country is considered a hantavirus-endemic area (10, 19). Since 2000, more than 250 cases have been reported with a cumulative fatality rate of 17% (20). As the patient studied in this clinical case was from that region, the clinicians’ first suspicion was HPS. The initial clinical history of this patient is compatible with HPS, including the urinalysis with leukocyturia and hematuria, as urinary tract infection has been described in the prodromal phase of this viral disease as well as in COVID-19 disease (21, 22). The patient presented transaminases slightly increased. However, the patient did not present thrombocytopenia nor hemoconcentration, which have been described during severe CHOV disease (10, 21). The patient had a rapid evolution towards an acute respiratory failure (24-48 hours after hospitalization) which is common with CHOV and other hantavirus infections (13, 23). On the other hand, respiratory failure generally presents later during COVID-19 disease progression (24). The decision to undertake differential diagnosis between SARS-CoV-2 and CHOV viruses was based on the March 9, 2020, epidemiological report, which included the first confirmed death caused by COVID-19 in Panama (3); this was 5 days before the patient´s development of acute respiratory failure.

It was crucial to confirm coinfection in this clinically complex case using molecular biology; diagnostic confirmation was obtained for SARS-CoV-2 and *Choclo orthohantavirus*. During the first months of the pandemic, Panama´s national protocol for COVID-19 management required two consecutive negative PCR for SARS-CoV-2 in patients at least 24 hours apart after >14 days of symptom onset, as a requirement for hospital discharge (16, 25). This was because SARS-CoV-2 PCR can be positive 46 days after infection (26), and at that moment of the COVID-19 pandemic, it was not clear if the detected viral genome corresponded to infectious virus (27, 28).

The humoral response to SARS-CoV-2 was detected before the response to CHOV. The rapid antibody response to SARS-CoV-2 seen in this patient was similar to what has been reported previously for COVID-19 (29). Generally, after hantavirus infection, virus-reactive IgM is rapidly generated in the acute phase, whereas IgG antibody titers rise more slowly in the convalescent phase (10, 30, 31). In contrast, in our patients’ sera, IgM antibodies against CHOV were not detected during the acute or convalescent phases, nonetheless, the presence of IgG antibodies was detected 30 days after the onset of symptoms. It is unknown if this could be due to the coinfection and the already activated response against SARS-CoV-2 (18). Neutralizing antibodies against SARS-CoV-2 were detected 11 days after the onset of symptoms in our patient; their titer decreased after one month and was high again 12 months after infection. It has been reported that around half of the patients with neutralizing activity lose or decrease the response after one month (32). After this initial rapid decrease, the neutralizing activity can persist for up to 6 months (33). For both viruses, the antibody response remained detected one year later, suggesting an extended humoral response as it has been reported previously (31).

The management of the patient in the early phase of the disease was done following Panama’s clinical protocol for CHOV infection and the indications at that moment for COVID-19 disease treatment (16, 20). Hydroxychloroquine was used as indicated, according to the evidence available at the beginning of the SARS-CoV-2 pandemic (16). However, since June 2020 WHO no longer recommended hydroxychloroquine due to the lack of robust evidence to support its use in COVID-19 management (34, 35). The patient was not treated with immunomodulators, corticosteroids, or ivermectin, which were other treatments been recommended worldwide for COVID-19 management, even if some of them, like ivermectin, have proved not useful (35).

The challenge in this patient is to know the precise moment when she was infected by each pathogen, considering that the incubation period to develop COVID-19 is from 2 to 21 days after SARS-CoV-2 infection and for hantavirus disease from 2 to 6 weeks after CHOV infection (13). Previous findings suggest that SARS-CoV-2 was already circulating in Panama by early-February 2020 (3). Thus, the clinical symptoms and the epidemiological studies could support the hypothesis that SARS-CoV-2 could have infected the patient between late February and the beginning of March 2020. The infection could have occurred also in the Soná District, where the patient lived. Additionally, the complete genome analysis evidenced a wide distribution of the virus in Panama, suggesting an intense cryptic SARS-CoV-2 transmission in the country that does not allow to precisely determine the moment or the possible place the infection occurred. The other important question is the moment of infection with CHOV. The patient living in the CHOV endemic area, commented that her next-door neighbor has bales of hay near the house, which may create a favorable condition for *Oligoryzomys fulvescens* (=*costaricensis*), the host reservoir of CHOV (11, 19).

This patient was case number 52 diagnosed with COVID-19 in Panama. Although Panama has over 20 years of experience in Hantavirus disease management, while COVID-19 is an emerging viral infection, the patient survived both pathologies with no long-term sequelae. PCR tests confirmed the diagnosis of coinfection, both agents were sequenced, and the presence of specific antibodies against SARS-CoV-2 and CHOV were determined. These antibodies were still detectable 12 months later. The detection of this coinfection between a new emergent virus and an endemic virus that emerged more than 20 years ago poses critical challenges in public health and differential diagnoses in the country. Other zoonoses in Panama that may require differential diagnosis with SARS-CoV-2 include Leptospirosis (36, 37) and Rickettsiosis (38).

The limitations of this clinical case description are the use of non-WHO-approved treatment like hydroxychloroquine and the fact that soluble cytokines were not analyzed in the patient because this was not part of the clinical management protocol at that time. The COVID-19 cluster analysis of this case allowed to show that the patient possibly transmitted SARS-CoV-2 at least to six direct contacts, thus it was not able to determine who infected her and the exact time of infection. Epidemiological cluster studies to detect other CHOV cases in the family and the neighbors were not done, nor capture of rodent reservoirs, due to the COVID-19 quarantine during which non-COVID-19 related field studies were forbidden. Moreover, the detection of neutralizing antibodies against CHOV could not be done as there is no protocol implemented yet. Finally, only a region of CHOV was sequenced because the viral load was too low for amplification of the whole S segment and no reagents were available for sequencing the complete genome. Nevertheless, this had no direct effect on the diagnosis and management of the patient.

During the pandemic in Arizona USA, Wilson et al. (2021) confirmed two fatal cases of HPS suspected of death by infection with SARS-CoV-2; one of them with a positive for SARS-CoV-2 RT-PCR from trachea and lung tissues without immunohistochemical assay positive result (14). This demonstrates carrying out highly specific and sensitive molecular diagnosis, to differentiate between infections with similar symptoms in a timely manner, during the acute phase of illness. As the production of antibodies is detected several days later, and some infections could cause cross-reactive issues during serological analyses (4, 39), diagnosis cannot depend solely on antibody detection. The use of new molecular diagnostic tools that can detect various pathogens at once is necessary. Therefore, molecular diagnostic capacity and genomic surveillance need to be increased throughout Central America to detect and alert about any eventual new emerging virus and increase rapid outbreak management.

## Patient Perspective

The patient assures that she fought for her life and was able to live thanks to the extraordinary work of the doctors and divine intervention; she was kept for 23 days in the ICU and 60 days in ward/hotel hospitalization fighting the two viruses.

## Data Availability Statement

The SARS-CoV-2 sequence of this study is available at GISAID (www.gisaid.org); accession number is EPI-ISL_1502818. The CHOV sequence of this study will be available at NCBI-GenBank (https://www.ncbi.nlm.nih.gov/genbank/); accession number is OK393713.

## Ethics Statement

Written informed consent was obtained from the individual for the publication of all the data included in this article.

## Author Contributions

SH, HN, EB, RC, ST, AC, IO, AJ, and CM attended the patients, collected the samples, and contributed to data acquisition. JRS, TPS, CG, OC, AM, JG, LÁ, YD, YP, DF, and MM-M analyzed the samples. JMP, AAM, and BA designed the study. SH, HN, CM, JRS, TPS, SL-V, AAM, BA, and JMP wrote and edited the manuscript. All authors read and approved the final manuscript.

## Funding

This work was supported by Project: “Study for the design of interventions for the prevention and control of hantavirus disease and other zoonoses” 111130409, 2020-2024 (BA) from the Ministry of Economy and Finance of Panama, the National laboratory COVID-19 response and National Research System (SNI), National Secretary of Research, Technology and Innovation (SENACYT).

## Conflict of Interest

The authors declare that the research was conducted in the absence of any commercial or financial relationships that could be construed as a potential conflict of interest.

## Publisher’s Note

All claims expressed in this article are solely those of the authors and do not necessarily represent those of their affiliated organizations, or those of the publisher, the editors and the reviewers. Any product that may be evaluated in this article, or claim that may be made by its manufacturer, is not guaranteed or endorsed by the publisher.

## Supplementary Material

Supplementary Figure 1 Map of Panama showing hantavirus pulmonary syndrome endemic area. HPS endemic area is delimited by a dashed line circle, including the region of Soná (dark gray). Hospitals (black circle) and Hospital-hotel (black square), in which the patient was managed are indicated.

## Abbreviations

COVID-19, coronavirus disease 2019; SARS-CoV-2, severe acute respiratory syndrome coronavirus 2; RT-PCR, real time reverse transcription polymerase chain reaction; HPS, Hantavirus Pulmonary Syndrome; CHOV, *Choclo orthohantavirus*.

## References

[1] Zhu N Zhang D Wang W Li X Yang B Song J. A Novel Coronavirus From Patients With Pneumonia in China, 2019. N Engl J Med (2020) 382(8):727–33. doi: 10.1056/NEJMoa2001017 PMC7092803 31978945

[2] Tang S Mao Y Jones RM Tan Q Ji JS Li N. Aerosol Transmission of SARS-CoV-2? Evidence, Prevention and Control Vol. 144. Environment International. Elsevier Ltd (2020). doi: 10.1016/j.envint.2020.106039 PMC7413047 32822927

[3] Franco D Gonzalez C Abrego LE Carrera JP Diaz Y Caicedo Y. Early Transmission Dynamics, Spread, and Genomic Characterization of SARS-CoV-2 in Panama. Emerging Infect Dis (2021) 27(2):612–5. doi: 10.3201/eid2702.203767 PMC7853578 33496228

[4] Masyeni S Santoso MS Widyaningsih PD Asmara DW Nainu F Harapan H. Serological Cross-Reaction and Coinfection of Dengue and COVID-19 in Asia: Experience From Indonesia. Int J Infect Dis (2021) 102:152–4. doi: 10.1016/j.ijid.2020.10.043 PMC7585717 33115680

[5] Bicudo N Bicudo E Costa JD Castro JALP Barra GB. Co-Infection of SARS-CoV-2 and Dengue Virus: A Clinical Challenge. Braz J Infect Dis (2020) 24(5):452–4. doi: 10.1016/j.bjid.2020.07.008 PMC7448779 32866435

[6] Díaz Y Chen-Germán M Quiroz E Carrera JP Cisneros J Moreno B. Molecular Epidemiology of Dengue in Panama: 25 Years of Circulation. Viruses (2019) 11(8):764. doi: 10.3390/v11080764 PMC6724401 31434193

[7] Armien B Pascale JM Bayard V Munoz C Mosca I Guerrero G. High Seroprevalence of Hantavirus Infection on the Azuero Peninsula of Panama. (2004). Available at: www.gorgas.gob.pa/docs/cuestionario%2007102003. 15211014

[8] Vincent MJ Quiroz E Gracia F Sanchez AJ Ksiazek TG Kitsutani PT. Hantavirus Pulmonary Syndrome in Panama: Identification of Novel Hantaviruses and Their Likely Reservoirs. Virology (2000) 277(1):14–9. doi: 10.1006/viro.2000.0563 11062031

[9] Nelson R Cañate R Pascale JM Dragoo JW Armien B Armien AG. Confirmation of Choclo Virus as the Cause of Hantavirus Cardiopulmonary Syndrome and High Serum Antibody Prevalence in Panama. J Med Virol (2010) 82(9):1586–93. doi: 10.1002/jmv.21864 PMC2927102 20648614

[10] Armien B Pascale JM Munoz C Marinas J Núnez H Herrera M. Hantavirus Fever Without Pulmonary Syndrome in Panama. Am J Trop Med Hyg (2013) 89(3):489–94. doi: 10.4269/ajtmh.12-0334 PMC3771286 23836565

[11] Ruedas LA Salazar-Bravo J Tinnin DS Armién B Cáceres L García A. Community Ecology of Small Mammal Populations in Panamá Following an Outbreak of Hantavirus Pulmonary Syndrome. J Vector Ecol (2004) 29(1):177–91. 15266755

[12] Armién AG Armién B Koster F Pascale JM Avila M Gonzalez P. Hantavirus Infection and Habitat Associations Among Rodent Populations in Agroecosystems of Panama: Implications for Human Disease Risk. Am J Trop Med Hyg (2009) 81(1):59–66. 19556568

[13] Jonsson CB Figueiredo LTM Vapalahti O. A Global Perspective on Hantavirus Ecology, Epidemiology, and Disease. Clin Microbiol Rev (2010) 23(2):412–41. doi: 10.1128/CMR.00062-09 PMC2863364 20375360

[14] Wilson TM Paddock CD Reagan-Steiner S Bhatnagar J Martines RB Wiens AL. Intersecting Paths of Emerging and Reemerging Infectious Diseases. Emerging Infect Dis (2021) 27(5):1517–9. doi: 10.3201/eid2705.204779 PMC8084486 33704045

[15] Hjelle B Jenison S Torrez-Martinez N Herring B Quan S Polito A. Rapid and Specific Detection of Sin Nombre Virus Antibodies in Patients With Hantavirus Pulmonary Syndrome by a Strip Immunoblot Assay Suitable for Field Diagnosis. J Clin Microbiol (1997) 35(3):600–8. doi: 10.1128/jcm.35.3.600-608.1997 PMC229635 9041397

[16] Tang LK Hevia E Pachar M Marquez F Quintero K Prado E. SMP V4 A Por La SocMP, MINSA M De S De P. Recomendaciones de Atención de Pacientes COVID-19 Hospitalizados 9 de Abril 2020 Sociedades Médicas de Panamá (2020). VERSIÓN 4.(49). Available at: http://minsa.b-cdn.net/sites/default/files/publicacion-general/recomendaciones_manejo_covid-19_version_4.0_9_de_abril_revisado_digesa_def14_abrildef.pdf.

[17] Atkinson B Jameson LJ Bovill BA Aarons EJ Clewlow J Lumley S. A Non-Fatal Case of Hantavirus Cardiopulmonary Syndrome Imported Into the UK (Ex Panama), July 2014. J Clin Virol (2015) 67:52–5. doi: 10.1016/j.jcv.2015.04.007 PMC4451477 25959159

[18] Feldman C Anderson R. The Role of Co-Infections and Secondary Infections in Patients With COVID-19. Pneumonia (Nathan Qld) (2021) 13(1):5. doi: 10.1186/s41479-021-00083-w 33894790 PMC8068564

[19] Armien B Pascale JM Munoz C Lee SJ Choi KL Avila M. Incidence Rate for Hantavirus Infections Without Pulmonary Syndrome, Panama. Emerg Infect Dis (2011) 17(10):1936–9. doi: 10.3201/eid1710.101717 PMC3310655 22000376

[20] Ministerio de Salud, Panamá. Guia De Manejo De La Enfermedad Por Hantavirus En Panamá. (2016). Available at: http://www.minsa.gob.pa/sites/default/files/publicacion-general/guia_hantavirus_0.pdf (Accessed October 15, 2021).

[21] Bayard V Kitsutani PT Barria EO Ruedas LA Tinnin DS Munoz C. Outbreak of Hantavirus Pulmonary Syndrome, Los Santos, Panama, 1999-2000. Emerg Infect (2004) 10(9):1635–42. doi: 10.3201/eid1009.040143 PMC3320309 15498167

[22] Chan L Chaudhary K Saha A Chauhan K Vaid A Zhao S. AKI in Hospitalized Patients With COVID-19. J Am Soc Nephrol (2021) 32(1):151–60. doi: 10.1681/ASN.2020050615 PMC7894657 32883700

[23] Duchin JS Koster FT Peters CJ Simpson GL Tempest B Zaki SR. Hantavirus Pulmonary Syndrome: A Clinical Description of 17 Patients With a Newly Recognized Disease. The Hantavirus Study Group. New Engl J Med (1994) 330(14):949–55. doi: 10.1056/NEJM199404073301401 8121458

[24] Li X Ma X. Acute Respiratory Failure in COVID-19: Is It “Typical” ARDS? Crit Care (2020) 24(1):1–5. doi: 10.1186/s13054-020-02911-9 32375845 PMC7202792

[25] WHO. Clinical Management of Severe Acute Respiratory Infection (SARI) When COVID-19 Disease Is Suspected: Interim Guidance, 13 March 2020. World Health Organization (2020). Available at: https://apps.who.int/iris/handle/10665/331446.

[26] Mallett S Allen AJ Graziadio S Taylor SA Sakai NS Green K. At What Times During Infection Is SARS-CoV-2 Detectable and No Longer Detectable Using RT-PCR-Based Tests? A Systematic Review of Individual Participant Data. BMC Med (2020) 18(1):346. doi: 10.1186/s12916-020-01810-8 33143712 PMC7609379

[27] NCIRD D of VD. Science Brief: Options to Reduce Quarantine for Contacts of Persons With SARS-CoV-2 Infection Using Symptom Monitoring and Diagnostic Testing. (2020). Available at: https://www.dshs.texas.gov/coronavirus/docs/CDCGuidanceonReducedQuarantine.pdf. 34009768

[28] Santacruz JV Ochoa MP Serrano JB Leon-Rojas JE. Criterios Para Definir a Un Paciente Como Recuperado_COVID19EC (2020). Available at: https://uanalisis.uide.edu.ec/cuales-son-los-criterios-para-definir-un-paciente-como-recuperado-de-covid-19/.

[29] Huang AT Garcia-Carreras B Hitchings MDT Yang B Katzelnick LC Rattigan SM. A Systematic Review of Antibody Mediated Immunity to Coronaviruses: Kinetics, Correlates of Protection, and Association With Severity. Nat Commun (2020) 11(1):1–16. doi: 10.1038/s41467-020-18450-4 32943637 PMC7499300

[30] Salinas TP Garrido JL Salazar JR Gonzalez P Zambrano N Fuentes-Villalobos F. Cytokine Profiles and Antibody Response Associated to Choclo Orthohantavirus Infection. Front Immunol (2021) 12(March):1–14. doi: 10.3389/fimmu.2021.603228 PMC8017165 33815363

[31] Krüger DH Schönrich G Klempa B. Human Pathogenic Hantaviruses and Prevention of Infection. Hum Vaccines (2011) 7(6):685–93. doi: 10.4161/hv.7.6.15197 PMC3219076 21508676

[32] Maeda K Higashi-Kuwata N Kinoshita N Kutsuna S Tsuchiya K. Hattori S Ichiro, Et al. Neutralization of SARS-CoV-2 With IgG From COVID-19-Convalescent Plasma. Sci Rep (2021) 11(1):5563. doi: 10.1038/s41598-021-84733-5 33692457 PMC7946899

[33] Pradenas E Trinité B Urrea V Marfil S Ávila-Nieto C Rodríguez de la Concepción ML. Stable Neutralizing Antibody Levels 6 Months After Mild and Severe COVID-19 Episodes. Med (2021) 2(3):313–320.e4. doi: 10.1016/j.medj.2021.01.005 33554155 PMC7847406

[34] Tang LK Hevia E Pachar M Marquez F Quintero K Prado E. Recomendaciones de Atención Intrahospitalaria de Pacientes COVID-19. Sociedades Médicas de Panamá (2020). Available at: https://www.spp.com.pa/wp-content/uploads/2021/01/Recomendaciones_manejo_intrahospitalario_COVID-19_Version_6.0.pdf.

[35] NIH. COVID-19 Treatment Guidelines 2. (2021). Available at: https://www.covid19treatmentguidelines.nih.gov/.

[36] Calero C Reinhard KR Owen CR. Leptospirosis of Man in the Isthmus of Panama. Am J Trop Med Hyg (1957) 6(6):1054–60. doi: 10.4269/ajtmh.1957.6.1054 13487978

[37] Alexander AD. The Distribution of Leptospirosis in Latin America. Bull World Health Organ (1960) 23(1):113. 13792576 PMC2555301

[38] Bermúdez SE Domínguez L Suárez JA Daza C Cumbrera A González J. Pasado y Presente de las Rickettsiosis en Panamaw. Panama: Instituto Conmemorativo Gorgas de Estudios de la Salud (2018). Available at: https://www.researchgate.net/publication/328639163_PASADO_Y_PRESENTE_DE_LAS_RICKETTSIOSIS_EN_PANAMA.

[39] Woodford J Sagara I Dicko A Zeguime A Doucoure M Kwan J. SARS-CoV-2 Seroassay Performance and Optimization in a Population With High Background Reactivity in Mali. J Infect Dis (2021) 6:jiab498. doi: 10.1093/infdis/jiab498 PMC8522418 34612499

