# Supplementary figures and images for: Case Report: First Confirmed Case of Coinfection of SARS-CoV-2 With Choclo orthohantavirus
Source: Front Trop Dis. 2021 Nov 10;2:769330. doi: 10.3389/fitd.2021.769330 (PMC8594034; doi:10.3389/fitd.2021.769330)

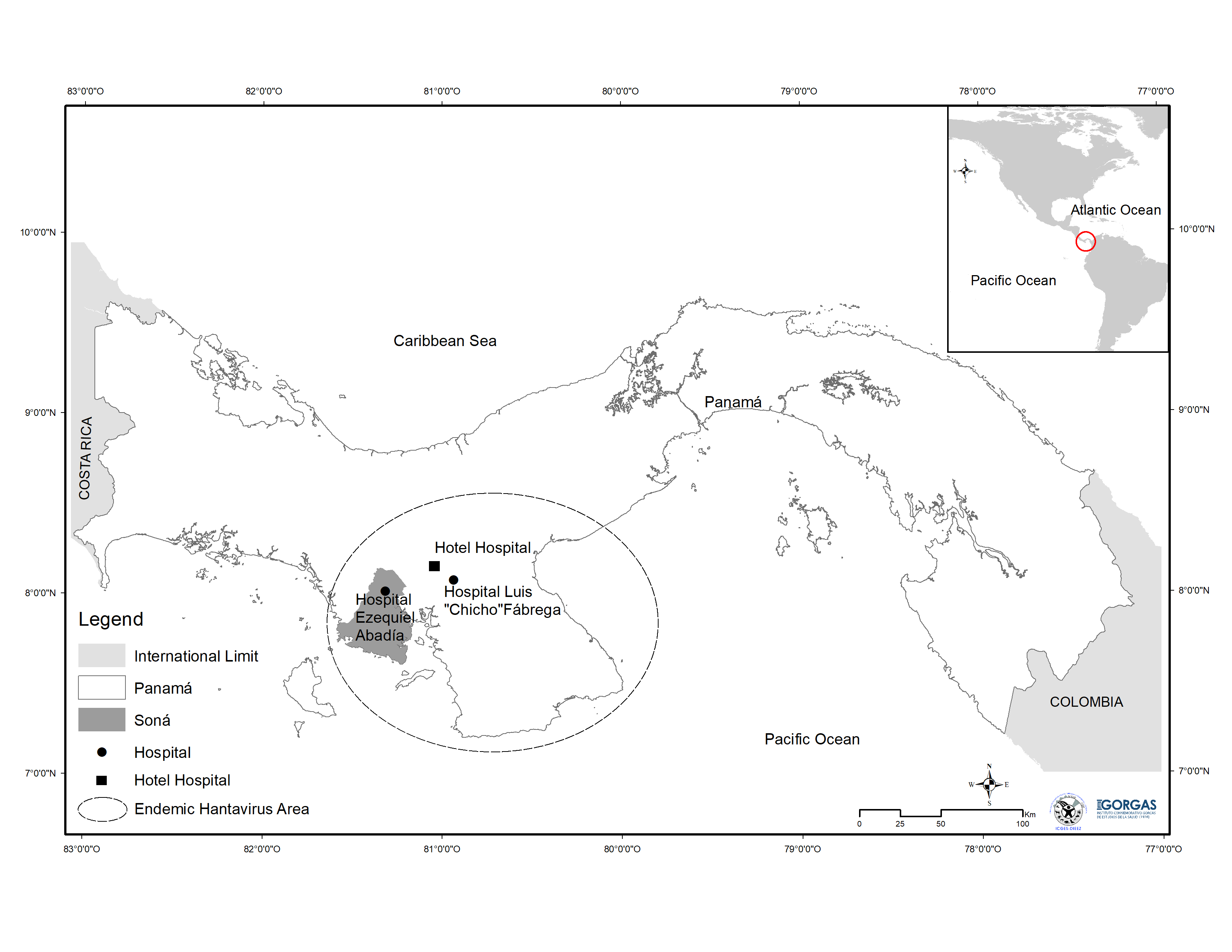

Supplement: Supplementary Figure 1 — Map of Panama showing hantavirus pulmonary syndrome endemic area. HPS endemic area is delimited by a dashed line circle, including the region of Soná (dark gray). Hospitals (black circle) and Hospital-hotel (black square), in which the patient was managed are indicated. [file Image_1.tif]
