# Supplementary material for: Case Report: First Confirmed Case of Coinfection of SARS-CoV-2 With Choclo orthohantavirus
Source: Front Trop Dis. 2021 Nov 10;2:769330. doi: 10.3389/fitd.2021.769330 (PMC8594034; doi:10.3389/fitd.2021.769330)
Supplement: Supplementary file 2 [file DataSheet_1.pdf]

Supplementary Material and Methods

Molecular diagnosis

First nasopharyngeal and oropharyngeal swab sample for SARS-CoV-2 detection was collected and send to Gorgas Memorial Institute for Health Studies. RNA was obtained using QiaAmp Viral RNA (Qiagen). Real time reverse transcription polymerase chain reaction (RT-PCR) for SARS-CoV-2 diagnosis was performed using Super Script III kit (Invitrogen-Thermo) and ABI 7500 Fast Real-Time PCR System (Applied Biosystem), as previously described (Franco et al., 2021). This sample was positive with a cycle threshold (Ct) value of 34.0. Posterior nasopharyngeal swab samples were collected and analyzed at the clinical laboratory (Molecular Section) of Luis "Chicho" Fabrega Hospital following manufacturer's instruction for automated ELITe InGenius system (ELITech Group).

The serum sample for detection of *Choclo orthohantavirus* (CHOV) was collected and send to Gorgas Memorial Institute for Health Studies. RNA was obtained using QiaAmp Viral RNA (Qiagen). Reverse transcription polymarase chain reaction (RT-PCR) and Nested PCR for CHOV diagnosis was performed using One Step RT-PCR and Taq PCR Master Mix kits (Qiagen), respectively, as previously described (Armien et al., 2013). An amplicon of 253 base pairs was obtained as expected.

Serological analysis

A total of six serum/plasma samples were obtained from the patient during acute illness from March 14 to April 6, 2020; and one sample in convalescent phase (April 5, 2021).

All samples were analyzed by enzyme immunoassay (EIA) for detection of immunoglobulin M (IgM) against CHOV, following manufacturer's instructions (Focus Diagnostics). Positive results should have an index value >1.1. Confirmation tests consists of strip immunoblot assay (SIA) for IgM and IgG detection against recombinant N protein of Hantavirus, processed as described by Hjelle *et al.*, 1997, in 1:100 and 1:200 dilution. Positive results may show a blue band marked on the nitrocellulose strip (Hjelle et al., 1997).

For detection of antibodies against SARS-CoV-2, chemiluminescent immunoassays (CLIA) using Virclia Monotest COVID-19 (Vircell Microbiologist) was performed following manufacturer's instructions. Positive results should have an index value >0.6 for IgA/IgM and >1.6 for IgG.

To titer neutralizing specific antibodies against SARS-CoV-2, 80% plaque reduction neutralization test (PRNT80) was performed on Vero cells (ATCC C1008) with SARS-CoV-2 Panamanian endemic lineage A.2.5. The assay was developed at the Gorgas Memorial Institute for Health Studies in BLS-3 laboratory facilities (Díaz et al., 2021).

Sequencing and molecular characterization of SARS-CoV-2

To perform an updated phylogeny of SARS-CoV-2, the analysis included local complete genome sequences obtained during the month of March through April 2020 (Franco et al., 2021) and genomes published worldwide in the GISAID data base after June 2020 but with collection dates until April 15, 2020. This analysis was performed using Nextstrain pipeline (Hadfield et al., 2018) and visualized with Baltic (https://github.com/evogytis/baltic.git) (SARS-CoV-2 sequence GISAID accession number: EPI-ISL_1502818)

Sequencing and molecular characterization of CHOV

Sanger sequencing reactions for CHOV were performed using ABI BigDye Terminator v3.1 Cycle Sequencing kit (Thermo Fisher Scientific) and Applied Biosystems 3130xl Genetic Analyzer (Thermo Fisher Scientific). Sequence identification using BLASTN 2.12.0+ program (Morgulis et al., 2008; Zhang et al., 2000). To evaluate the phylogenetic relation of the obtained sequence with others previous sequenced *Choclo orthohantavirus* strains and reference Hantavirus from elsewhere, a maximum likelihood tree was calculated using IQ-TREE v. 2.0.3 (Minh et al., 2020), the best substitution model was chose using ModelFinder (Kalyaanamoorthy et al., 2017) and the node support was calculated using ultrafast bootstrap (Thi Hoang et al., 2017). Consensus tree generated was draw using FigTree (http://tree.bio.ed.ac.uk/software/figtree/) (*Choclo orthohantavirus* sequence GenBank accession number: OK393713)

References

Armien, B., Pascale, J.M., Munoz, C., Marinas, J., Núnez, H., Herrera, M., Trujillo, J., Sánchez, D., Mendoza, Y., Hjelle, B., Koser, F., 2013. Hantavirus fever without pulmonary syndrome in panama. American Journal of Tropical Medicine and Hygiene 89, 489–494. https://doi.org/10.4269/ajtmh.12-0334

Díaz, Y., Ortiz, A., Weeden, A., Castillo, D., González, C., Moreno, B., Martínez-Montero, M., Castillo, M., Vasquez, G., Sáenz, L., Franco, D., Pitti, Y., Chavarria, O., Gondola, J., Moreno, A.M., Ábrego, L., Beltrán, D., Guerra, I., Chang, J., Chaverra, Z., Guerrero, I., Valoy, A., Gaitán, M., Araúz, D., Morán, E., Chen-Germán, M., Valdespino, E., Rodríguez, R., Corrales, R., Chen-Camaño, R., Pascale, J.M., Martínez, A.A., López-Vergès, S., 2021. SARS-CoV-2 reinfection with a virus harboring mutation in the Spike and the Nucleocapsid proteins in Panama. International Journal of Infectious Diseases 108, 588–591. https://doi.org/10.1016/j.ijid.2021.06.004

Franco, D., Gonzalez, C., Abrego, L.E., Carrera, J.P., Diaz, Y., Caicedo, Y., Moreno, A., Chavarria, O., Gondola, J., Castillo, M., Valdespino, E., Gaitán, M., Martínez-Mandiche, J., Hayer, L., Gonzalez, P., Lange, C., Molto, Y., Mojica, D., Ramos, R., Mastelari, M., Cerezo, L., Moreno, L., Donnelly, C.A., Pascale, J.M., Faria, N.R., Lopez-Verges, S., Martinez, A.A., 2021. Early transmission dynamics, spread, and genomic characterization of SARS-CoV-2 in Panama. Emerging Infectious Diseases 27, 612–615. https://doi.org/10.3201/eid2702.203767

Hadfield, J., Megill, C., Bell, S.M., Huddleston, J., Potter, B., Callender, C., Sagulenko, P., Bedford, T., Neher, R.A., 2018. NextStrain: Real-time tracking of pathogen evolution. Bioinformatics 34, 4121–4123. https://doi.org/10.1093/bioinformatics/bty407

Hjelle, B., Jenison, S., Torrez-Martinez, N., Herring, B., Quan, S., Polito, A., Pichuantes, S., Yamada, T., Morris, C., Elgh, F., Lee, H.W., Artsob, H., Dinello, R., 1997. Rapid and specific detection of Sin Nombre virus antibodies in patients with hantavirus pulmonary syndrome by a strip immunoblot assay suitable for field diagnosis. Journal of Clinical Microbiology 35, 600–608. https://doi.org/10.1128/jcm.35.3.600-608.1997

Kalyaanamoorthy, S., Minh, B.Q., Wong, T.K.F., von Haeseler, A., Jermiin, L.S., 2017. ModelFinder: Fast model selection for accurate phylogenetic estimates. Nature Methods 14, 587–589. https://doi.org/10.1038/nmeth.4285

Minh, B.Q., Schmidt, H.A., Chernomor, O., Schrempf, D., Woodhams, M.D., von Haeseler, A., Lanfear, R., Teeling, E., 2020. IQ-TREE 2: New Models and Efficient Methods for Phylogenetic Inference in the Genomic Era. Molecular Biology and Evolution 37, 1530–1534. https://doi.org/10.1093/molbev/msaa015

Morgulis, A., Coulouris, G., Raytselis, Y., Madden, T.L., Agarwala, R., Schäffer, A.A., 2008. Database indexing for production MegaBLAST searches. Bioinformatics 24, 1757–1764. https://doi.org/10.1093/bioinformatics/btn322

Thi Hoang, D., Chernomor, O., von Haeseler, A., Quang Minh, B., Sy Vinh, L., Rosenberg, M.S., 2017. UFBoot2: Improving the Ultrafast Bootstrap Approximation. Mol. Biol. Evol 35, 518–522. https://doi.org/10.5281/zenodo.854445

Zhang, Z., Schwartz, S., Wagner, L., Miller, W., 2000. A greedy algorithm for aligning DNA sequences. Journal of computational biology : a journal of computational molecular cell biology 7, 203–214. https://doi.org/10.1089/10665270050081478
